# The harmonic ratio of trunk acceleration predicts falling among older people: results of a 1-year prospective study

**DOI:** 10.1186/1743-0003-10-7

**Published:** 2013-01-28

**Authors:** Takehiko Doi, Soichiro Hirata, Rei Ono, Kota Tsutsumimoto, Shogo Misu, Hiroshi Ando

**Affiliations:** 1 Section for Health Promotion, Department for Research and Development to Support Independent Life of Elderly, Center for Gerontology and Social Science, National Center for Geriatrics and Gerontology, 35 Gengo Morioka, Obu, Aichi, 474-8511, Japan; 2 Department of Rehabilitation Science, Kobe University Graduate School of Health Sciences, 7-10-2 Sumaku Tomogaoka, Kobe, Hyogo, Japan; 3 Higashihiroshima Orthopedic Clinic, Higashihiroshima Clinic Building, 4281-1 Saijochomisono, Higashiroshima, Hiroshima, Japan; 4 Department of Community Health Sciences, Kobe University Graduate School of Health Sciences, 7-10-2 Sumaku Tomogaoka, Kobe, Hyogo, Japan

**Keywords:** Trunk, Gait, Acceleration, Falls

## Abstract

**Background:**

Gait variables derived from trunk accelerometry may predict the risk of falls; however, their associations with falls are not fully understood. The purpose of the study was to determine which gait variables derived from upper and lower trunk accelerometry are associated with the incidence of falls, and to compare the discriminative ability of gait variables and physical performance.

**Methods:**

This study was a 1-year prospective study. Older people (*n* = 73) walked normally while wearing accelerometers attached to the upper and lower trunk. Participants were classified as fallers (*n* = 16) or non-fallers (*n* = 57) based on the incidence of falls over 1 year. The harmonic ratio (HR) of the upper and lower trunk was measured. Physical performance was measured in five chair stands and in the timed up and go test.

**Results:**

The HR of the upper and lower trunk were consistently lower in fallers than non-fallers (*P* < 0.05). Upper trunk HR, was independently associated with the incidence of falls (*P* < 0.05) after adjusting for confounding factors including physical performances. Consequently, upper trunk HR showed high discrimination for the risk of falls (AUC = 0.81).

**Conclusions:**

HR derived from upper trunk accelerometry may predict the risk of falls, independently of physical performance. The discriminative ability of HR for the risk of falls may have some validity, and further studies are needed to confirm the clinical relevance of trunk HR.

## Background

Falls are relatively common among older people and about one-third of people aged ≥ 65 years fall at least once a year [1,2]. The progressive decline in physical performance and mobility is a major risk factor for falling, and physical tests are used to evaluate the risk of falling. Physical performance also serves as a target for interventions aimed at preventing falls [2]. Nevertheless, some older individuals may have a normal gait [3], while others have characteristic gait features caused by age-related changes. Gait variables that have been characterised in various clinical conditions among older people include frailty [4] and cognitive decline [5], and are also associated with survival [6].

Trunk movement during walking plays a critical role in successful locomotion and contributes to gait stability among older people [7]. One variable used to evaluate trunk movement during walking is the smoothness of trunk acceleration, which declines progressively with age [8-10]. The smoothness of trunk acceleration during walking was also suggestive of gait dysfunction [11,12] or the risk of falls in a cross-sectional study [13]. To date, however, few prospective studies have investigated the association between trunk smoothness and falls among older people. Furthermore, trunk acceleration measured in different locations (i.e., lower and upper trunk) varied among studies. One of the roles of the trunk during walking is to attenuate oscillations, decreasing their frequency from the lower to upper trunk, which helps to stabilise the head and gait [14-16]. In this way, the trunk filters the acceleration signal from the lower to the upper trunk during gait, allowing us to determine differences in acceleration signals between specific locations of the trunk. Although the difference in acceleration between the upper and lower trunk may influence the relationship between trunk acceleration and fall, it is not clear whether the smoothness of acceleration in the lower or upper trunk is more clinically relevant for assessing the risk of falling.

Therefore, the aim of this study was to determine the clinical relevance of gait variables, derived from upper and lower trunk accelerometry, on the risk of falls. We conducted a 1-year prospective study to determine which gait variables derived from trunk acceleration are associated with the risk of falling among older people. To examine the ability of gait variables to predict falling, we compared the predictive abilities of gait variables with those of physical tests that are clinically used to assess the risk of falling.

## Methods

### Study participants

Ninety three community-dwelling older people, aged ≥ 65 years, were recruited through a community association centre providing services for older people who were living in the community. People with a history of serious neurological diagnoses that clearly affected gait, such as Parkinson disease, were excluded. People with adequate hearing, vision and speech who were capable of participating in the clinical examinations, and who could walk independently were eligible for this study. Seventeen three people (mean age: 80.8 years, 57 women) met the criteria and participated in this study. Demographic data, including age, sex, height, weight and body mass index were recorded. Cognitive function was evaluated using the Mini-Mental State Examination [17]. The participants’ medical condition was determined at interviews and the presence of major diseases was recorded. The Research Ethics Committee of the Kobe University Graduate School of Medicine approved the study (approval number 901). Written informed consent was obtained from all participants in accordance with the Declaration of Helsinki.

### Fall assessment

The incidence of fall was a primary outcome in this prospective study. A fall was defined as an unexpected event in which the participant came to rest on the ground, floor or lower level [18]. We excluded falls derived from extraordinary environmental factors (e.g., fall from a ladder). Falls were recorded for 12 months after the initial gait experiments and other assessments. Participants were instructed to record all falls and research assistants collected information about falls at least once a week in face-to-face interviews and at group meetings held at the community association centre. Participants who experienced a fall at least once during the follow-up period were classified as a faller, while the other participants were classified as non-faller.

### Physical performance

Physical function was assessed by five chair stands (FCS) and the timed up and go test (TUG). In the FCS, participants were required to stand up and sit down five times as quickly as possible, and the time taken was used as the FCS score [19]. The TUG is a mobility test in which the participants were asked to walk 3 m then turn around and walk 3 m at their self-selected normal pace in a well-lit environment [20].

### Gait procedure and apparatus

The gait studies were conducted on a 15-m smooth, horizontal walkway, with a 2.5-m space before each end of the walkway for acceleration and deceleration. The participants were instructed to walk at a normal pace and measurements were performed over the medial 10-m distance. Gait analysis was done using accelerometers that are already used in clinical settings because accelerometers do not restrict the participants’ movements, and are less expensive than other equipment [21]. The apparatus setting used for gait analysis is reported in more detail elsewhere [22]. In brief, trunk movement was measured using tri-axial accelerometers (MA3-04AC, MicroStone Co., Nagano, Japan) attached to the C7 spinous process (upper trunk) and the L3 spinous process (lower trunk), using a Velcro™ belt and surgical tape without restricting the movement of the subject. Trunk linear accelerations were measured along the vertical (VT), anteroposterior (AP) and mediolateral (ML) axes, sampled at 200 Hz, and all acceleration signals were synchronised. The accelerometers were calibrated before starting measurements. After analogue to digital transformation, the signals were collected in a data logger and immediately transferred to a laptop personal computer (Vaio VGN, Sony Co., Tokyo, Japan) via a Bluetooth Personal Area Network. The working range of the accelerometer to the laptop was approximately 50 m.

### Data processing

Signal processing was performed using commercially available software (MATLAB, Release 2008, Math Works Inc., Natick, MA, USA). The person who processed the data was blinded to the subjects’ characteristics. Before analysis, all acceleration data were low-pass filtered (dual pass zero lag Butterworth filter) with a cutoff frequency of 20 Hz. Each stride was detected as previously reported [23] as the interval from an initial contact event to the next ipsilateral event. The harmonic ratio (HR) was determined separately in all three directions (HR-VT, MR-ML and HR-AP) and was analysed as described elsewhere. In brief, the HR was computed using digital Fourier transformation in each direction individually. The HR represents the smoothness and stability of trunk movement during gait [10,24]. A higher HR indicates smoother and more stable trunk movement during gait.

### Statistical analysis

All analyses were performed using SPSS 19 for Windows (SPSS Inc., Chicago, IL, USA). We conducted statistical analyses after confirming the data were normally distributed using the Shapiro-Wilk test. We compared the characteristics and gait variables between fallers and non-fallers using independent *t*-tests or χ^2^ tests (Table 1). Logistic regression analysis was used to examine the association between the incidence of falls and each gait variable. In this analysis, the incidence of falls was used as the dependent variable while independent variables included gait variables and physical performance as continuous measures. Confounding factors were selected as those that were significantly different between fallers and non-fallers at *P* < 0.05 in bivariate analyses. The final logistic regression model was developed by forward stepwise selection from all variables that were significantly associated with falling in bivariate analyses (*P* < 0.05). Receiver operating characteristic (ROC) curves were used to estimate the cutoff values for gait variables to predict the future incidence of falling, focusing on the gait variables that were significantly associated with falling in the logistic regression analyses. The area under the curve (AUC) was calculated from the ROC curve for each variable and the cut-off value was calculated based on the Youden index [25]. Statistical significance was set at *P* < 0.05.

**Table 1:** Subject characteristics

**Variables**	**Fallers *n *= 16**	**Non-fallers *n *= 57**	***P *value**
Age, years	84.8 ± 5.9	79.7 ± 8.2	0.022
Sex (female), %	94	74	0.168
Height, m	1.47 ± 0.10	1.51 ± 0.10	0.162
Weight, kg	48.3 ± 9.0	53.0 ± 9.7	0.089
Body mass index, kg/m^2^	22.4 ± 2.9	23.2 ± 3.1	0.342
Mini-Mental State Examination, score	24 ± 4	25 ± 5	0.423
Medical conditions			
Osteoarthritis/rheumatism, %	27	14	0.258
Diabetes mellitus, %	20	14	0.687
Hypertension, %	40	49	0.574
Heart disease, %	20	14	0.687
Number of diseases	1.6 ± 0.8	1.6 ± 1.0	0.840
Number of medications used	2.9 ± 3.8	3.2 ± 3.7	0.691

Values are means ± standard deviation or percentages. *P* values were calculated using independent *t*-tests or χ^2^ tests.

## Results

The characteristics of the participants are summarised in Table 1. Participants were classified as fallers (i.e., those who fell at least once during the 1-year study; *n* = 16, 22%) or as non-fallers (*n* = 57, 78%). Four participants fell multiple times, and the mean numbers of falls among fallers was 1.4. Age was significantly different between the two groups, whereas other characteristics, including medical history and cognitive function, were not.

Fallers walked significantly more slowly than non-fallers (fallers: 0.63 ± 0.27 m/s, non-fallers: 0.98 ± 0.34 m/s, *P* < 0.001). The HR data for both groups are presented in Table 2. HR-VT and HR-AP, but not HR-ML, of the upper trunk were significantly lower in fallers than in non-fallers (HR-VT: *P* < 0.001; HR-ML: *P* = 0.308; HR-AP: *P* = 0.005). HR-VT, HR-ML and HR-AP of the lower trunk were also significantly lower in fallers than in non-fallers (HR-VT: *P* = 0.013; HR-ML: *P* = 0.035; HR-AP: *P* = 0.018). Physical performance was better in non-fallers, as FCS (fallers: 19.6 ± 9.4 s; non-fallers: 13.8 ± 5.7 m/s; *P* = 0.037) and TUG (fallers: 20.7 ± 10.6 s; non-fallers: 14.1 ± 7.5 s; *P* = 0.031) were both longer in fallers than in non-fallers.

**Table 2:** Comparison of gait variables between fallers and non-fallers

**Variables**	**Fallers *n *= 16**	**Non-fallers *n *= 57**	***P *value**
Walking speed, m/s	0.63 ± 0.27	0.98 ± 0.34	< 0.001
Upper trunk			
HR-VT	1.84 ± 0.63	2.71 ± 0.87	< 0.001
HR-ML	2.16 ± 1.02	2.44 ± 0.93	0.308
HR-AP	1.69 ± 0.92	2.56 ± 1.11	0.005
Lower trunk			
HR-VT	2.07 ± 0.64	2.69 ± 0.93	0.013
HR-ML	1.49 ± 0.35	1.89 ± 0.73	0.035
HR-AP	2.00 ± 0.90	2.67 ± 0.99	0.018

Values are means ± standard deviation. *P* values were calculated using independent *t*-tests. HR: harmonic ratio; VT: vertical; ML: mediolateral; AP: anteroposterior.

**Figure 1:**
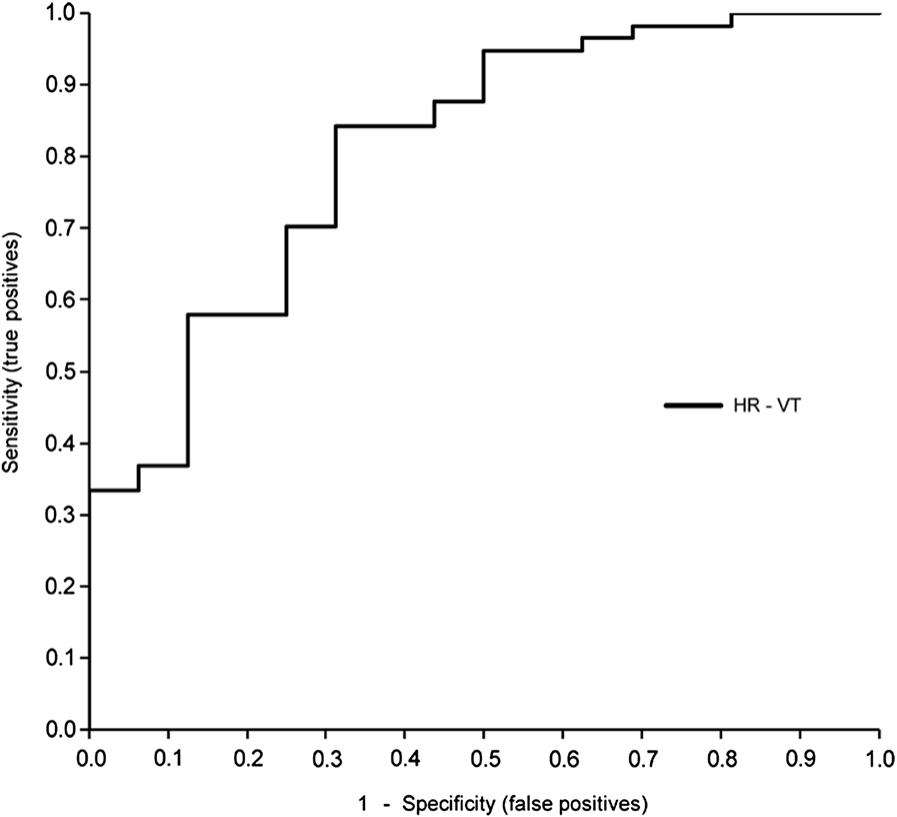
**Receiver operator characteristic (ROC) curves of the harmonic ratio (HR) of upper trunk acceleration in the vertical direction (VT) to predict the incidence of falls. **The area under the ROC curve for HR-VT is 0.81.

The results of logistic regression analysis are shown in Table 3. In bivariate logistic analyses, HR and physical performance were significantly associated with the incidence of falling (FCS: *P* = 0.016; TUG: *P* = 0.013; WS: *P* = 0.002). Using stepwise selection, HR-VT of the upper trunk was a significant predictor of falling (odds ratio: 0.24, *P* = 0.026). The AUC for HR-VT of the upper trunk was 0.81 (95% CI: 0.69–0.93; *P* < 0.001) (Figure 1). The cutoff value calculated for HR-VT, based on the Youden index, was 1.89 (specificity, 84.2%; sensitivity, 68.8%).

**Table 3:** Falls, physical performance tests and gait variables

**Variables**	**Model 1**		**Model 2**	
	**OR (95% CI)**	***P *value**	**OR (95% CI)**	***P *value**
Five chair stands	1.12 (1.02–1.22)	0.016		
Timed up and go test	1.08 (1.02–1.15)	0.013	1.11 (0.98–1.25)	0.092
Walking speed	0.02 (0.001–0.23)	0.002		
Upper trunk				
HR-VT	0.16 (0.06–0.49)	0.001	0.24 (0.07–0.84)	0.026
HR-ML	0.69 (0.34–1.40)	0.304		
HR-AP	0.31 (0.13–0.73)	0.007		
Lower trunk				
HR-VT	0.35 (0.15–0.83)	0.017		
HR-ML	0.24 (0.06–0.95)	0.042		
HR-AP	0.43 (0.21–0.90)	0.024		

Logistic regression analyses were conducted with fall/non-fall as the dependent variable. Physical performance scores and gait parameters derived from trunk acceleration were included as independent variables. Model 1 shows the crude odds ratios obtained in bivariate analyses for each independent variable. Model 2 was developed by stepwise variable selection. OR: odds ratio; CI: confidence interval; HR: harmonic ratio; VT: vertical; ML: mediolateral; AP: anteroposterior.

## Discussion

Our results indicate that gait variables derived from trunk accelerometry provide good discrimination of the risk of falling among older people. The HR values of the upper and lower trunk were lower in fallers than in non-fallers. Physical performance (i.e., the results of the FCS and TUG tests) was also inferior in fallers. The associations between the HR of the upper trunk and the risk of falling were independent, even in the regression model that included physical performance. By contrast the association between the HR of the lower trunk and the risk of falling was weakened in this model, although the HR of the lower trunk was lower in all directions compared with the corresponding HR of the upper trunk. The HR of the upper trunk showed directional characteristics in that the HR in the VT and AP direction, but not in the ML direction, were significantly lower in fallers, and only HR in the VT direction of the upper trunk was a significant predictor of falls. ROC curve analysis confirmed that the HR of the upper trunk showed high specificity for detecting the risk of future falls.

Measurements obtained from accelerometry showed good clinical usefulness and ability to detect the risk of falls among older people in several studies [21,26]. As an outcome of gait analysis, the HR of trunk acceleration represents the smoothness of trunk movement during gait, and its association with falling was confirmed in a cross-sectional study [13]. Our prospective study extends these earlier results and confirms the usefulness of measuring trunk acceleration to assess the risk of falling. Our results are consistent with earlier reports, as the fallers had lower HRs of the upper and lower trunk than non-fallers [13]. Furthermore, the HR of the upper trunk were significantly associated with the incidence of falls, even after adjusting for confounding variables, and showed high discriminative values (AUC = 0.81). By comparison, the associations between the HR of the lower trunk and falls were weakened in the regression model. Trunk movement during walking plays important roles in filtering and attenuating oscillations to stabilise the head [14-16]. The amplitude of acceleration and signal regularity are filtered from the lower trunk to the upper trunk, and the postural system maintained within the trunk segment contributes to overall stability [14]. In fact, the deterioration in the HR was greater in fallers than in non-fallers, suggesting that the trunk’s role as a filter may be impaired in fallers. This role of the trunk may lead to the differing associations of HRs between the upper and lower trunk with the risk of falls.

Similar associations between gait variables derived from trunk accelerometry, physical performance and risk of falls to those observed in our study were reported in another study [27]. Bautmans *et al.*[27] analysed lower trunk acceleration during walking and investigated its relationship with physical performance and the risk of falls using the autocorrelation procedure. They suggested that variability of lower trunk acceleration was correlated with physical performance and that the discrimination of the risk of falls, based on trunk variability, decreased after taking into account walking speed. Our results show similarities in that the variables derived from trunk accelerometry and the relationship between lower trunk variables and the risk of falls was attenuated in the regression model including confounding factors, such as walking speed, although the methods used to processed data and measure trunk acceleration differed between the two studies. Physical performance scores on the FCS and TUG tests were also associated with the incidence of falling in bivariate analysis, while regression analysis suggested that the HR may have an advantage over physical performance for assessing the risk of falling. These results suggest that gait analysis should be incorporated into assessments of the risk of falling and that enhanced trunk stability may lead to robust physical function and thus prevent falls.

The HR shows directional differences across studies investigating the HR of trunk acceleration during gait [8-10,13,14]. For example, Mentz *et al.* reported that participants at high risk of falling walked with decreasing HR in all directions of lower trunk acceleration, and in the VT and AP directions of head acceleration [13]. Our results are consistent with these directional characteristics of the HR in relation to falls. Indeed, the HR of the lower trunk in all directions and the HR of the upper trunk in the VT and AP directions were lower in the fallers than in the non-fallers in our study. Our results also revealed that the HR of the upper trunk in the VT direction may represent the risk of falling. The physical characteristics of older people vary greatly, and ranged from very fit to frail in earlier studies, so differences in physical function among older people should be taken into account when determining the risk of falling and when implementing interventions aimed at reducing the incidence of falling [2]. The participants in our study were not physically fit (the mean walking speed was 0.91 m/s), similar to the people at high risk of falling in the earlier studies that used accelerometry [13,27]. Furthermore, the fallers in our study were generally older and walked more slowly (mean walking speed: 0.63) than the non-fallers. Considering the differences in physical performance between studies, large cohort studies of older people across the spectrum of physical fitness are needed to better understand the association between trunk acceleration and risk of falls.

Our study had several limitations that should be discussed. First, the number of participants was relatively small, as was the number of multiple fallers, which prevented us from examining the associations between multiple falls and gait variables. Multiple falls is associated with a high risk of injury and post-fall syndrome [2]. Thus, further studies are needed to examine the association between multiple falls and trunk acceleration. Second, other factors may confound the risk of falling, particularly cognitive function and trunk alignment. These potential confounders may also interact with aging. Although the MMSE was used to assess general cognitive function, more detailed domains, focusing on executive function and/or memory, should also be assessed. These cognitive functions may affect the outcome of a fall. Fall is a multifactorial outcome and further studies focusing on several factors, including cognitive function and trunk alignment, are needed to investigate the associations between falling and gait variables derived from trunk acceleration. Finally, the incidence of falling in our subjects was relatively low compared with that in other studies [1,2], while a recent systematic review estimated that the incidence of falls among older people ranged from 14.7% to 34% [28]. These differences may be due to differences between races and/or physical function status of the participants. Clearly, a large cohort study is needed to determine the incidence of falls among older adults.

## Conclusions

In conclusion, the HR of trunk acceleration showed good discriminative ability to predict the incidence of falls among older people. Gait analysis using an accelerometer may be a useful tool to assess the risk of falling among older people. To generalise the clinical relevance of gait variables derived from trunk accelerometry, large cohort studies of participants across a broader spectrum of clinical characteristics are needed.

## Abbreviations

FCS: Five chair stands; TUG: Timed up and go test; VT: Vertical; AP: Anteroposterior; ML: Mediolateral; HR: Harmonic ratio; ROC: Receiver operating characteristic; AUC: Area under the curve.

## Competing interests

The authors declare that they have no competing interests.

## Authors’ contributions

TD and RO substantially contributed to the conception of the methods used and helped to write the manuscript. KT and SM were involved in the acquisition, analysis and interpretation of data. SH and HA substantially contributed to the conception and design of the study, analysis and interpretation of the data, and helped to write the manuscript. All authors read and approved the final manuscript.
